# Detection of REEs with lightweight UAV-based hyperspectral imaging

**DOI:** 10.1038/s41598-020-74422-0

**Published:** 2020-10-15

**Authors:** René Booysen, Robert Jackisch, Sandra Lorenz, Robert Zimmermann, Moritz Kirsch, Paul A. M. Nex, Richard Gloaguen

**Affiliations:** 1 Helmholtz-Zentrum Dresden-Rossendorf, Helmholtz Institute Freiberg for Resource Technology, 09599 Freiberg, Germany; 2 School of Geosciences, University of the Witwatersrand, Johannesburg, 2000 South Africa

**Keywords:** Environmental impact, Solid Earth sciences, Economic geology

## Abstract

Rare earth elements (REEs) supply is important to ensure the energy transition, e-mobility and ultimately to achieve the sustainable development goals of the United Nations. Conventional exploration techniques usually rely on substantial geological field work including dense in-situ sampling with long delays until provision of analytical results. However, this approach is limited by land accessibility, financial status, climate and public opposition. Efficient and innovative methods are required to mitigate these limitations. The use of lightweight unmanned aerial vehicles (UAVs) provides a unique opportunity to conduct rapid and non-invasive exploration even in socially sensitive areas and in relatively inaccessible locations. We employ drones with hyperspectral sensors to detect REEs at the earth’s surface and thus contribute to a rapidly evolving field at the cutting edge of exploration technologies. We showcase for the first time the direct mapping of REEs with lightweight hyperspectral UAV platforms. Our solution has the advantage of quick turn-around times (< 1 d), low detection limits (< 200 ppm for Nd) and is ideally suited to support exploration campaigns. This procedure was successfully tested and validated in two areas: Marinkas Quellen, Namibia, and Siilinjärvi, Finland. This strategy should invigorate the use of drones in exploration and for the monitoring of mining activities.

## Introduction

Sustainable exploration and mining of REEs are required to implement the energy transition, e-mobility and enable the economic growth of society. Independent and ethical supply of critical raw materials has been strongly encouraged in Europe^1^ as well as in the U.S.A.^2^. Despite several exploration initiatives all around the world, only a few REE projects (e.g., Browns Range, Australia) reached the extraction stage during the last years. Within this context, we recently proposed a novel approach for the exploration of REEs applying remote sensing techniques^3,4^.

REE deposits can be divided into two general categories. Primary deposits are associated with magmatic processes and hydrothermal fluid mobilisation and precipitation whereas secondary deposits originate from the movement of REE minerals from their place of origin, such as through sedimentary concentration and weathering^5^. Five main types of REE deposits are currently exploited; (1) carbonatites, (2) magmatic alkaline deposits, (3) ionic clay deposits, (4) laterite deposits and (5) placer deposits.

Currently, the most important source of REEs are carbonatite deposits and their associated alkaline igneous rocks^6^. Carbonatites are magmatic rocks with > 50 modal-% carbonate content^7^. In nature, the 17 REEs are commonly found together due to them sharing a trivalent charge and similar ionic radii^8^. Reflective spectroscopy can be used to identify various surficial rock forming minerals as well as rare earths. Neodymium (Nd) has some of the most pronounced absorption features among the REEs and therefore can be used as a key pathfinder element for REEs as a whole^9^. Nd has characteristic absorption features in the Visible to Near-Infrared (VNIR) range of the electromagnetic spectrum at  580 nm,  750 nm and  800 nm^10^. Turner et al.^11^, Boesche et al.^12^ and Neave et al.^9^ successfully identified and mapped REEs using laboratory- or ground-based hyperspectral imaging (HSI). Booysen et al.^3^ demonstrated how unmanned aerial vehicle (UAV)-based hyperspectral data can be used in a multi-scale remote sensing exploration approach to map REEs indirectly. Minute spectral features, low concentrations, large ground sampling distances (GSDs) and heavy imaging equipment have impeded their direct measurements from lightweight airborne platforms so far.

Drones have been increasingly used in mineral exploration (e.g., Booysen et al.^3^; Jackisch et al.^13^; Malehmir et al.^14^; Sayab et al.^15^). Difficult terrain or outcrops unreachable on foot or by vehicles can be rapidly surveyed with UAVs from a safe distance with minimal personnel on site, ensuring safety, efficiency and speed^16,17^. A few studies have shown the potential of using UAV-based HSI for geological mapping (e.g., Jackisch et al.^13^). In the context of carbonate geology, Madjid et al.^18^ and Dujoncquoy et al.^19^ used UAV-based photogrammetry in order to map sedimentary carbonate lithologies. However, none of these studies have directly investigated carbonatite-hosted REE exploration with UAV-based HSI yet. We now suggest to test the potential of UAV-based HSI to detect REEs directly as a consequence of the recent improvements in lightweight HSI sensors.

**Figure 1:**
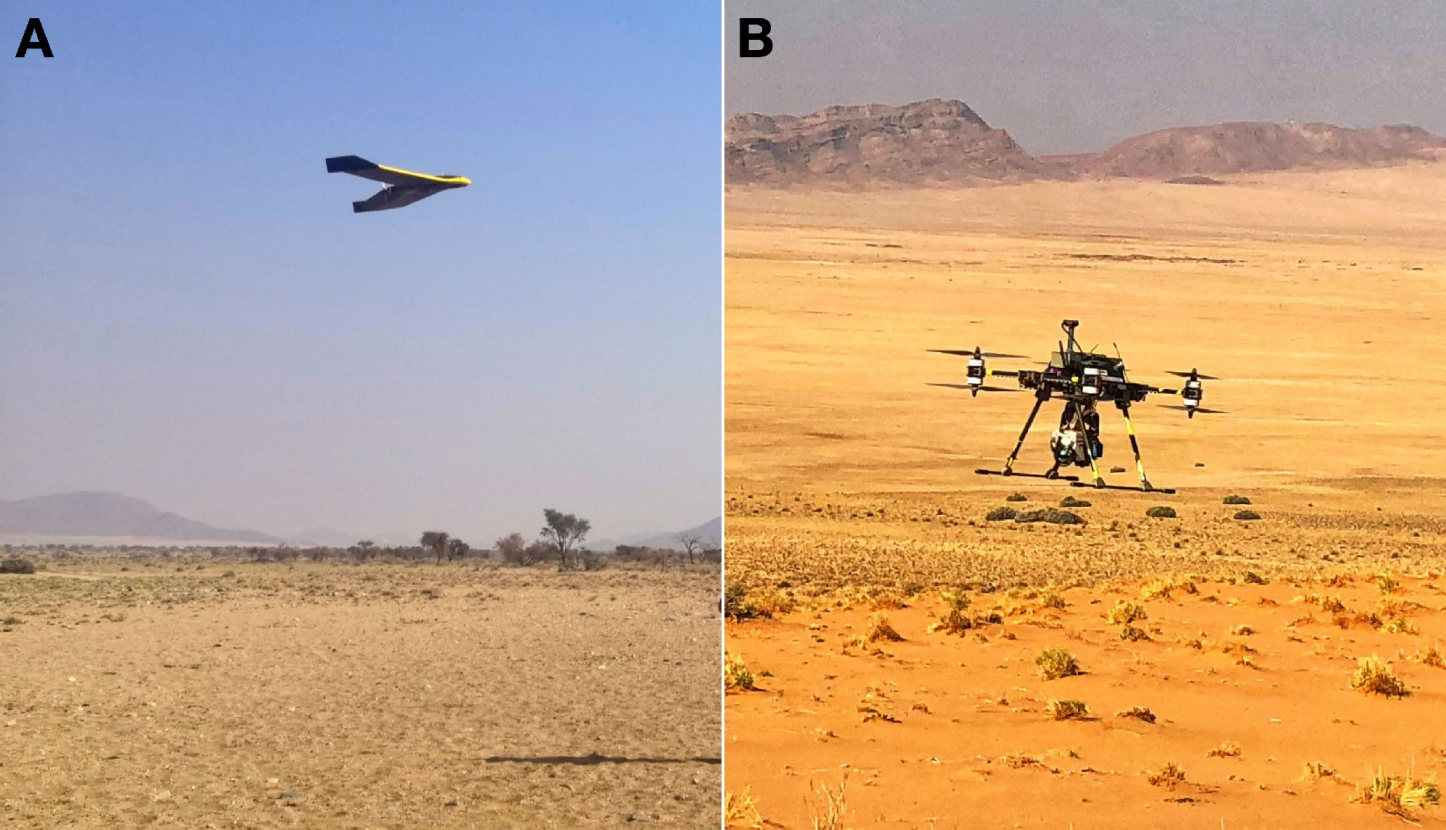
(**A**) Fixed-winged system: RGB camera attached to a senseFly eBeePlus. (**B**) Multi-rotor system: Rikola sensor attached to a Tholeg THO-R-PX-8/10.

We have recently proposed an innovative approach for the acquisition and processing of UAV-based HSI data^3,20^. We use two types of unmanned aerial systems (UAS); a fixed-wing system (Fig. 1A) for the rapid acquisition of photogrammetric data as basis for digital surface models and a multi-copter (Fig. 1B) for HSI collection. Using a fixed-winged system, we capture nadir stereo-imagery with a portable snapshot camera, also referred as RGB camera as it acquires three bands in the visible part of the electromagnetic spectrum (Red, Green and Blue) for Structure-from-Motion Multi-Vision-Stereo (SfM-MVS) photogrammetry. With this technique, we rapidly produce high spatial resolution digital surface models (DSMs) and orthomosaics that are used to correct the drone-borne hyperspectral images. Producing the DSMs and orthomosaics can take up to a few hours depending of the data size using a standard laptop (e.g., i7 processor with 16 GB RAM). We capture hyperspectral data with a frame-based sensor, the Senop Rikola Hyperspectral Imager in this case, mounted on a multi-rotor platform. Additionally, we take in-situ spectral measurements with a portable VNIR-SWIR field spectrometer and samples for petrological and geochemical validation.

In this paper we aim to identify and directly map the characteristic Nd absorption features with drone-borne hyperspectral data in carbonatite bodies. The objectives are (1) to develop a fast, operational and reliable drone-borne hyperspectral procedure to identify areas with high REE concentrations and (2) test the practicality of using drones in the exploration of REEs; i.e., can it be easily deployed and integrated into the exploration process. We illustrate our approach in two carbonatite complexes to demonstrate the versatility of using drones in different climatic regions, one in a subarctic region, the Siilinjärvi phosphate mine in Finland, and the other in an arid environment, the Marinkas Quellen complex in Namibia.

**Figure 2:**
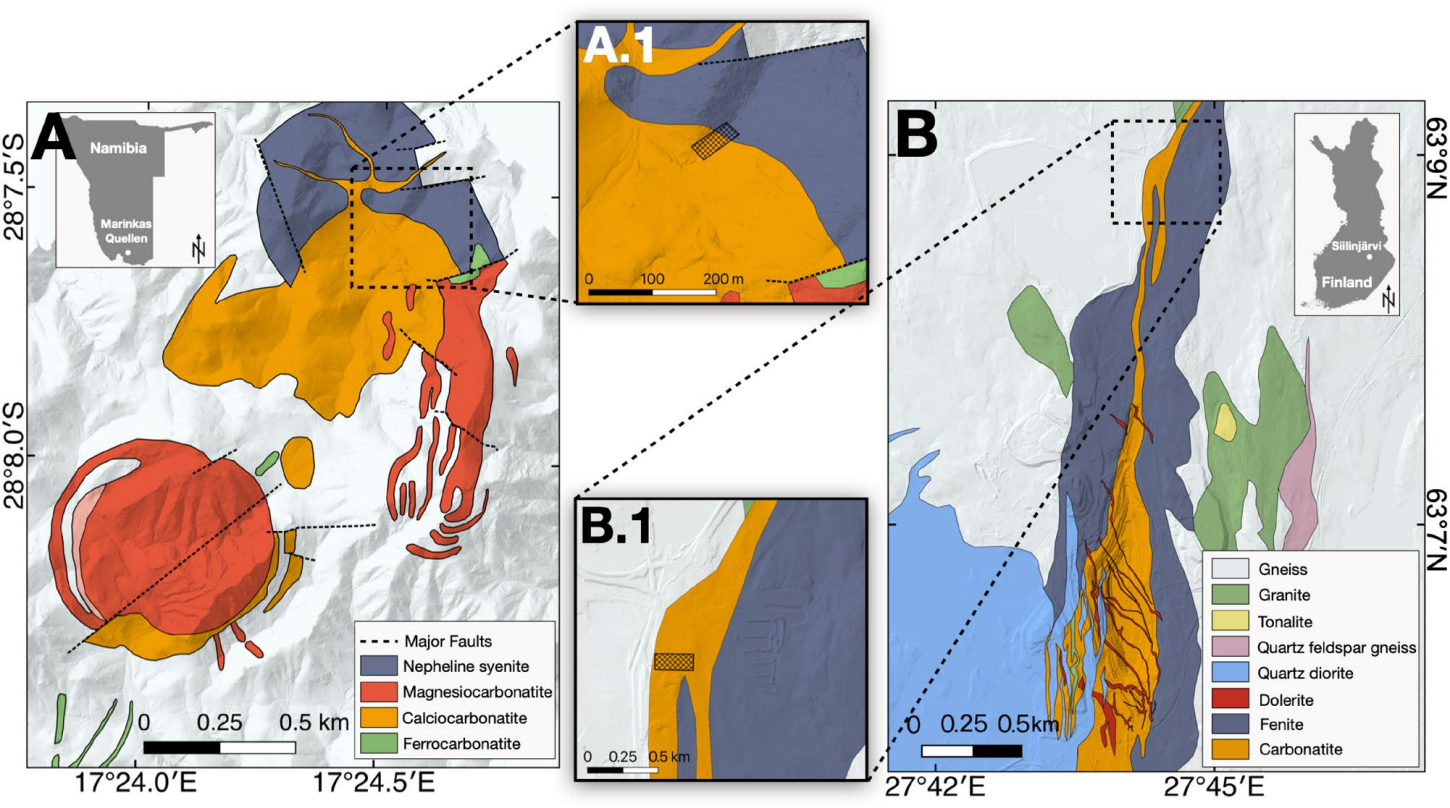
(**A**) Geological map of the Marinkas Quellen carbonatite complex with an inset showing its location in Namibia. The geological map was modified after Smithies and Marsh^22^. (**A.1**) The zoomed-in area shows the footprint of the hyperspectral UAV-based survey. (**B**) Geological map of the Siilinjärvi carbonatite complex with an inset showing its location in Finland. The geological map was modified after the Geological Survey of Finland^26^. (**B.1**) The zoomed in area shows the footprint of the hyperspectral UAV-based survey. Both maps were fused with a digital elevation model (DEM) created with photogrammetry from own UAV-based data and produced in QGIS 3.12.

We chose these two representative sites to highlight the diversity of environments in which REEs can be found:

- The Marinkas Quellen alkaline-carbonatite intrusive complex is located near the border of South Africa in southern Namibia, in a very arid and remote part of the desert. Most of the complex is not accessible by car and demands long hikes to access the target areas and thus typical of green-field sites. It is part of the 490–550 Ma Kuboos-Bremen Igneous Province^21^ and is composed of alkali-granites, syenites and carbonatites^22^. The carbonatites at Marinkas Quellen range from calcio-, magnesio- to ferro-types (Fig. 2A). Late ferrocarbonatite dykes show enrichment in manganese, REEs, and thorium^22^.
- The Archean Siilinjärvi carbonatite complex in central Finland is one of the oldest known carbonatites^23^ and hosts the only operating phosphorous mine in the European Union. The site is situated near the Arctic Circle but is easily accessible by car. This site represents a typical brown-field exploration site. The mineral of interest, apatite, is associated with a glimmerite-carbonatite intrusion that forms up to a 1.5 km thick, steeply dipping tabular lens, which is surrounded by fenitized basement gneisses. It is intruded by tonalites and multiple generations of basalt dikes^24^ (Fig. 2B). Although not currently exploited as an REE-resource, the main lithologies at Siilinjärvi contain REE-bearing monazite, apatite, pyrochlore, strontianite, and titanium–niobium-phases^24,25^. The exposed outcrops at these two sites have little to no vegetation cover that makes them ideal settings to showcase our UAV-based approach.

## Results

**Figure 3:**
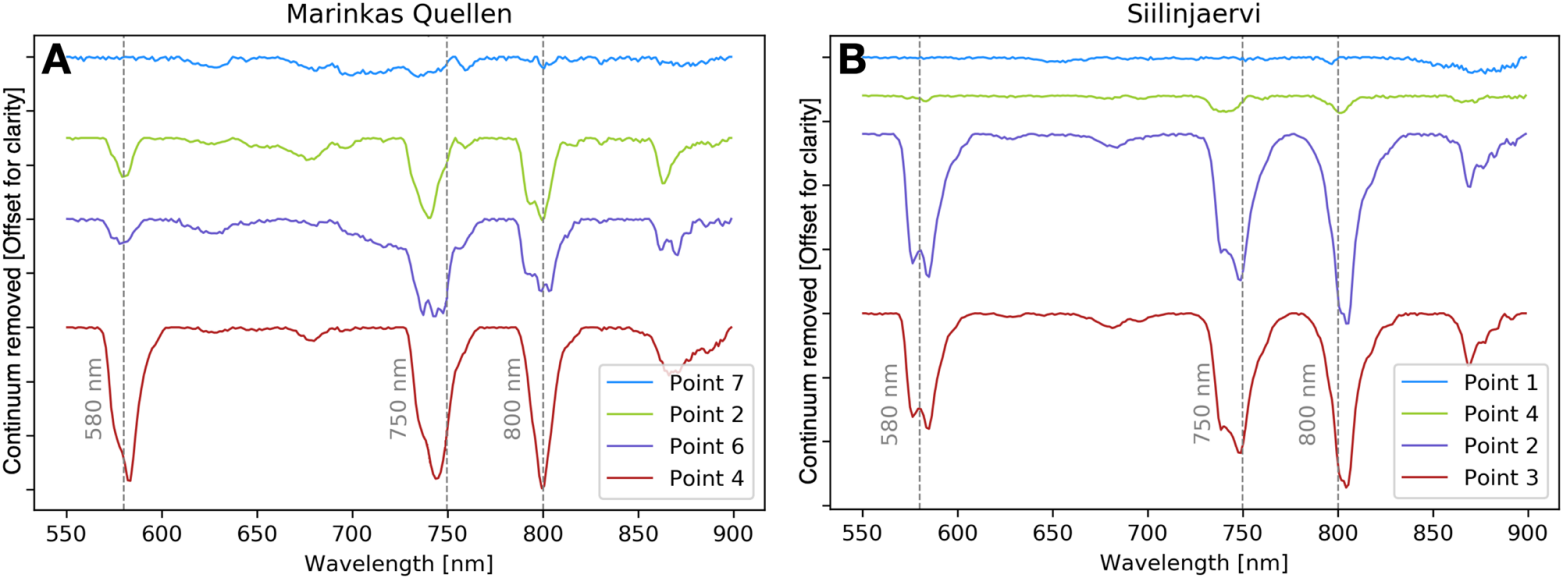
(**A**) Graph showing the reflectance of in-situ handheld spectra from selected points in Marinkas Quellen indicated on Fig. 4. (**B**) Graph showing the reflectance of in-situ handheld spectra from selected points in Siilinjärvi indicated on Fig. 5.

**Figure 4:**
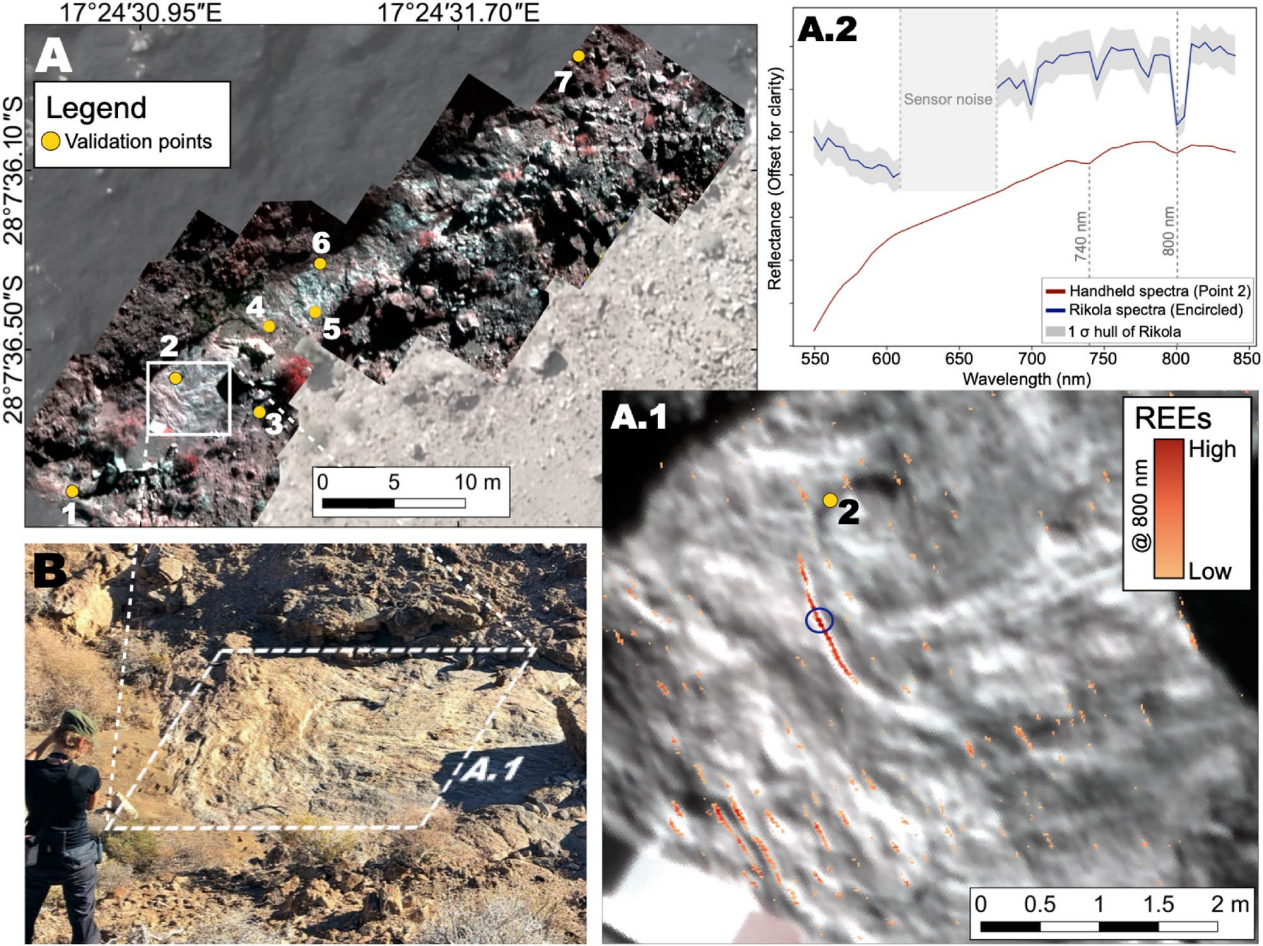
Marinkas Quellen. (**A**) UAV-based hyperspectral mosaic overlaying high-resolution UAV-based orthomosaic created in QGIS 3.12 (own data). The locations of in-situ measurements taken for validation are shown in yellow. (**A.1**) Close-up of the HSI data superposed with pixels mapped with 800 nm absorption feature (yellow to red indicating the increasing depth of the absorption feature i.e., high = very deep, low = shallow). The acquisition location of spectra shown in A.2 is represented by a blue circle and point 2. (**A.2**) Comparison of averaged Rikola with handheld spectra. (**B**) Field photo showing the area selected for Nd mapping.

**Figure 5:**
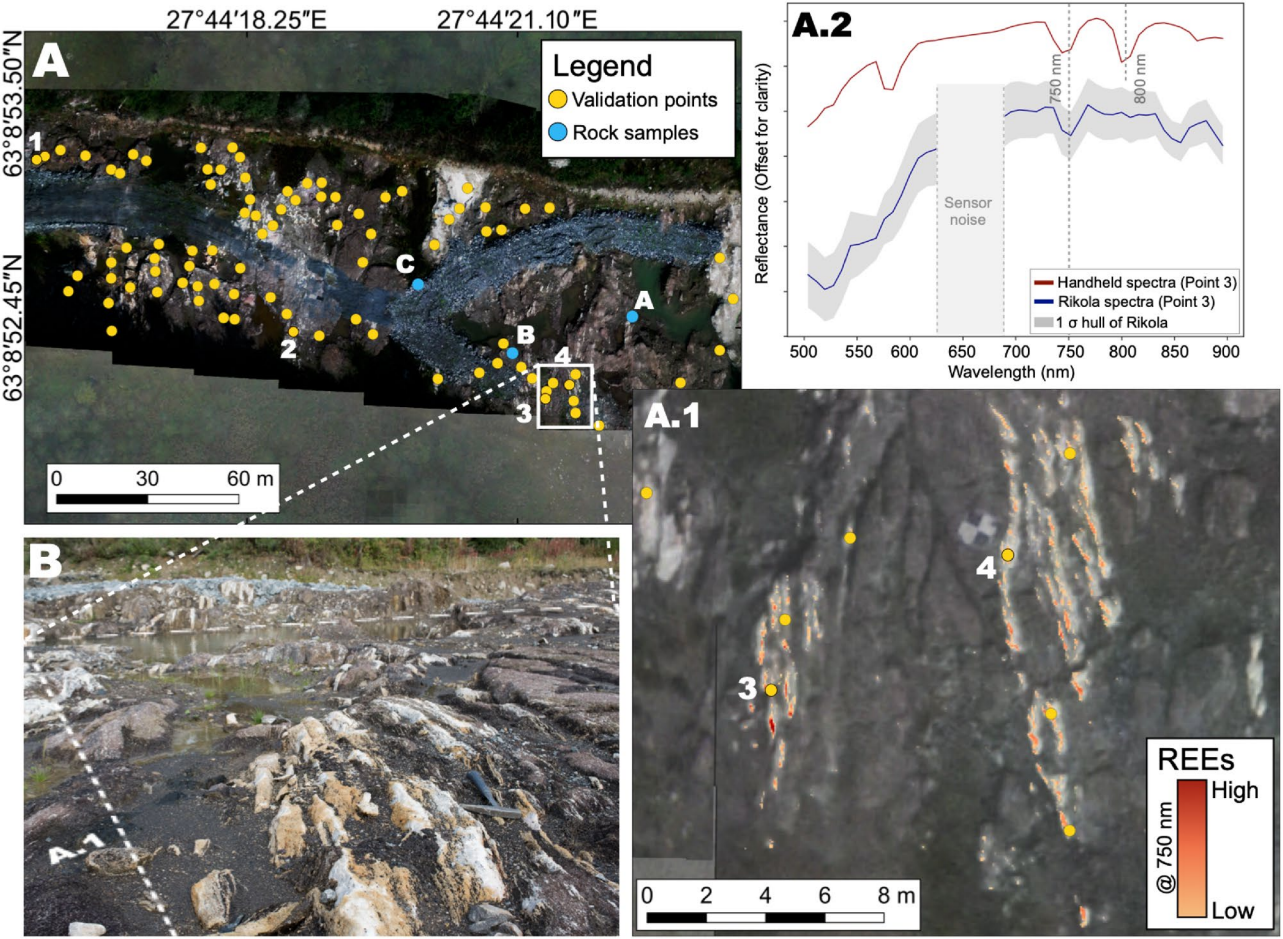
Siilinjärvi. (**A**) UAV-based hyperspectral mosaic overlaying the high-resolution UAV-based orthomosaic created in QGIS 3.12 (own data). The locations of in-situ measurements taken for validation are shown in yellow and blue. (**A.1**) Close-up of the HSI data superposed with pixels mapped with 750 nm absorption feature (yellow to red indicating the increasing depth of the absorption feature i.e., high = very deep, low = shallow). The acquisition location of spectra shown in A.2 is represented by point 3. (**A.2**) Comparison of averaged Rikola with handheld spectra. (**B**) Field photo illustrating the area selected for Nd mapping.

We highlight the results of the two UAV-based hyperspectral surveys in Namibia and Finland in the following section. We selected areas of interest of approximately 10,000 m^2^ based on preliminary field campaigns. We took 7 in-situ spectral measurements and sampled those same spots at Marinkas Quellen as well as > 80 spectral measurements and 3 samples from Siilinjärvi. We selected the amount of measurements and samples locations at each site based on the size of the area, feasibility (e.g., accessibility and exposure) and representativeness. We attempted to space out the sampling spots evenly throughout the outcrops and selected spots not covered by vegetation. These measurements and samples were used to validate the data obtained from UAVs and to confirm the locations of possible REE enriched zones in the outcrops. Four representative spectra of these measurements from each case study are shown in Fig. 3A,B, their locations are indicated on Figs. 4 and 5.

Inductively coupled plasma mass spectrometry (ICP-MS) analysis confirmed that samples taken from locations 2, 4 and 6 from Marinkas Quellen contained between 260 and 650 ppm Nd as well as 0.18 and 0.57% Total Rare Earth Oxides (TREO) while location 7 contained much less at 0.02% TREO (Table 1). Spectral measurements determined that locations 2, 4 and 6 also contained REEs as they showed typical spectral absorption features of Nd at 580 nm, 750 nm and 800 nm whereas location 7 did not display any REE characteristics. At Siilinjärvi, only the spectra at locations 2 and 3 showed prominent REE absorption features at the three aforementioned wavelengths. Location 4 displays only very weak Nd signatures. The ICP-MS results showed that samples taken from the same relative locations (locations A, B and C) contained between 65 and 325 ppm Nd as well as 0.03 and 0.18 % TREO (Table 2). Additionally, the absorption features at Siilinjärvi were noticeably deeper than at Marinkas Quellen. The double absorption features at 740 and 750 nm seen at Siilinjärvi at locations 2 and 3, and at location 6 of Marinkas Quellen can be attributed to a typical Dysprosium (Dy) absorption feature close to Nd^10^.

We captured UAV-based hyperspectral data over the carbonatite outcrops at a relative flight altitude of 40 m for the Siilinjärvi site and 20 m for the Marinkas Quellen area, resulting in a ground sampling distance (GSD) of 3 cm and 1.5 cm respectively. These altitudes were chosen to balance area coverage and spatial resolution. The pre-processed data were co-registered to the RGB orthomosaic in order to create hyperspectral mosaics of each area of interest (Figs. 4, 5A). After calibration and validation, we mapped the occurrence of the most prominent feature identified in the HSI data using minimum wavelength mapping (MWM), at 800 nm in the case study at Marinkas Quellen and at 750 nm in Siilinjärvi. The possible reasons for the different positions of the most conspicuous REE absorption components between the two sites is discussed in the following section.

**Table 1:** ICP-MS results of samples taken from Marinkas Quellen, indicating the amount of Nd and Total Rare Earth Oxides (TREO).

Location	Nd (ppm)	TREO (%)
Point 1	225	0.16
Point 2	278	0.18
Point 3	443	0.29
Point 4	261	0.20
Point 5	130	0.09
Point 6	647	0.57
Point 7	19	0.02

**Table 2:** ICP-MS results of samples taken from Siilinjärvi, indicating the amount of Nd and Total Rare Earth Oxides (TREO).

Location	Nd (ppm)	TREO (%)
Point A	67	0.03
Point B	157	0.09
Point C	325	0.18

The MWM maps are displayed in Figs. 4A.1 and 5A.1 respectively. Field and high-resolution photo observations indicate that the mapped REEs are spatially coherent and preponderantly located in the carbonatite bodies and not in the surrounding country rock in both cases. In Marinkas Quellen, the REEs seem to be mostly concentrated in a lenticular structure located in the centre of the outcrop (Fig. 4A.1) and on a set of parallel NW-SE trending stripes, oblique to the sensor matrix. In Siilinjärvi, the REEs are located in elongated carbonatite bodies with very little to no REEs mapped in the surrounding rock (Fig. 5A.1).

**Figure 6:**
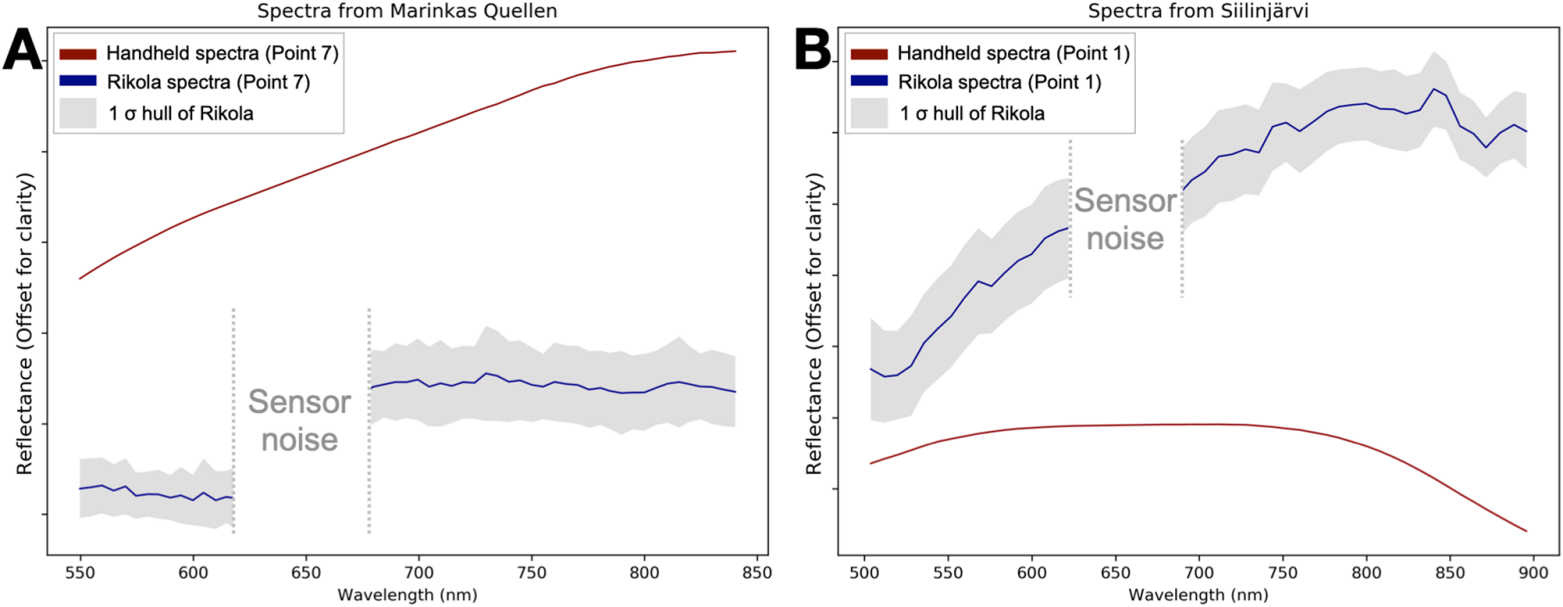
(**A**) Graph showing handheld spectra against Rikola spectra taken at the same location at Marinkas Quellen indicating the absence of Nd absorption features. (**B**) Graph showing handheld spectra against Rikola spectra taken at the same location at Siilinjärvi indicating the absence of Nd absorption features.

To illustrate the accuracy of the REE maps, we compare in-situ spectra with their homologous spectra extracted from the HSI data (Figs. 4, 5A.2). The range marked as sensor noise seen in Figs. 4A.2 and 5A.2 is caused by a known technical issue at  640 nm in the Rikola imager that does not affect the rest of the spectrum^27^. Additionally, we plot the spectral signatures at locations showing no absorption features indicating the absence of REEs (Point 7 from Marinkas Quellen and point 1 from Siilinjärvi). These control locations (Figs. 6A,B) display homologue spectra on the HSI data that also lack the typical Nd absorption features.

## Discussion

We observe that we can map the prominent absorption features of Nd from flight heights reaching 40 m above ground with a lightweight snapshot HSI camera. To validate the accuracy of the sensor, we first tested the hyperspectral imager in laboratory conditions. The results showed that it is possible to detect the narrow absorption features of Nd at 580 nm, 740 nm and 800 nm by selecting a spectral sampling interval of 5 nm and a resolution of 15 nm^3^. However, a slight spectral shift can occur depending on the spectral resolution, which needs to be accounted for when performing MWM. In addition, despite the potential ability to detect absorption features at any spectral position, this ability is limited by the sensor settings. In operational mode, the sensor can record 50 pre-determined bands. The sampling choice has an effect on the detection of narrow absorption features. For example, the 800 nm absorption feature was not prominent in the data of Siilinjärvi, thus we mapped the 750 nm feature. The difficulty in resolving this feature was most probably caused by inadequate spectral sampling. The data from Marinkas Quellen had a spectral sampling of 5 nm around 800 nm while it was 8 nm for the data acquired in Siilinjärvi. The acquisition strategy in Marinkas Quellen was adapted to REE mapping with an adapted spectral interval while in Siilinjärvi the survey was more general with a default spectral sampling. It is likely that the spectral sampling was too broad and offset to effectively pick-up the narrow absorption feature typically found at around 800 nm in Siilinjärvi. On the whole, the spatial coherence of the detected REEs demonstrates that the mapping is accurate. The spatial accuracy is supported by a spectral consistency at the expected absorption position attested by histograms of the pixel wavelengths for each data set (Fig. 8A,B).

The results shown in this study demonstrate that we can directly map REE occurrences with UAV-based hyperspectral data under certain conditions, mainly using Nd as a proxy for total REEs. From our study we identified five main requirements that may pose a challenge, however we can address and solve most issues with a dedicated approach. These requirements include:

1. Having an uncovered and sufficiently illuminated outcrop:
  We can target outcrops without vegetation cover and capture data when the sun is highest resulting in a sub-nadir viewing and sub-vertical illumination.
2. Adequate REE concentrations:
  With the advancements in sensor and platform technology and improvements in processing algorithms, the accurate mapping of REEs can now be accessible to those not specialized in remote sensing. Rowan et al.^28^ proposed that 300 ppm Nd is sufficient to produce absorption features using a spectrometer in the laboratory. We were able to detect Nd absorption features at locations where whole rock Nd concentrations are as low as 130 ppm at Marinkas Quellen and 160 ppm Nd in Siilinjärvi. On the other hand, there is no clear relationship between whole rock Nd concentrations and spectral feature depths. This can be partly explained by the crystal sizes of the REE-bearing minerals. The REE bearing minerals, i.e. apatites, are quite large in Siilinjärvi whereas they are typically small in the sampled areas of Marinkas Quellen. The lowest concentrations of REEs required for detection with drone-borne HSI can thus vary from deposit to deposit.
3. Adequate sensor settings:
  Hyperspectral data with a maximum spectral sampling intervals of 5 to 8 nm with a spectral resolution (full width at half maximum (FWHM)) of at least 15 nm is required.
4. A robust processing workflow for UAV-based hyperspectral surveys:
  Software to correct for relief, atmospheric and adjacency effects, and to orthorectify hyperspectral drone-borne data. In addition, as the use of UAVs is becoming more popular in geology, it is paramount that standardised pre-processing methods are developed to ensure well-corrected, quality data^29^.
5. Reasonable weather conditions:
  Lastly, in order to conduct any successful drone-borne survey there has to be little to no rain or snow, and wind speeds typically below 12 m/s

In this study, we investigated the pertinence of exploring REE occurrences using an innovative and non-invasive UAV-based hyperspectral methodology. The typical and current approach to geological mapping in mineral exploration is a combination of surface geological mapping with surficial and/or trench sampling at specific intervals^30^. The specimens are then sent to laboratories for whole-rock geochemical analysis. The mapping and geochemical analyses occur before drilling and can be costly and time-consuming^31^. Our solution can give the field geologist a rapid estimation of relative REE distribution and thus improve the sampling strategy by identifying zones of high REE concentrations within the rock outcrops. In a wider sense, UAV-based hyperspectral surveys offer greater flexibility to the geologist on site. Certain areas might be inaccessible due to rough terrain, steep slopes, proneness to rock falls or dense vegetation, but can now be surveyed from a safe distance to create lithological maps and/or determine whether those areas need further investigation. Our study also demonstrated that we surveyed two test sites, one in an arid environment and the other in subarctic conditions and were able to map REEs successfully. This indicates that we can improve field work by accelerating field mapping by identifying zones of interest the day of acquisition and not wait for laboratory analyses in two contrasting, harsh environments.

With all the benefits of UAVs in REE exploration, there are still a few limiting factors. Uncovered outcrops are needed to accurately detect mineralization, and soil and vegetation covers are a major limitation in any geological remote sensing survey and can not be circumvented by the use of UAVs either. Strong winds (> 12 m/s) are another limiting factor in the use of lightweight UAVs. As with any high-tech equipment, faulty hardware, weak GNSS signal in mountainous areas or inefficient access to a power grid to charge batteries can impede field activities and cause delays. In addition, the area that can be covered by a UAV-based survey is much smaller than satellite or plane-based solutions. Bearing in mind these limiting factors, our work indicates that UAVs can enhance mapping and precision targeting of REEs on a local scale at a very high spatial resolution in many situations. Besides providing a very high spatial resolution, the use of UAVs can overcome some other problems encountered when using satellite or plane-based data. Clouds are typical obstacles that obscure the Earth’s surface in satellite and strongly limit plane-based acquisitions. The flexibility of lightweight UAVs implies that they can be deployed easily and whenever needed without having to wait for scenes to be acquired or commissioned. The presented solution is not intended to replace ground surveys but to allow for a more detailed targeting of REEs that will require fewer invasive geological activities, increase the overall safety of the field workers and accelerate the general process.

New sensor technologies that capture spectral data in the shortwave infrared (SWIR) range will allow an even better direct targeting of host rock mineralogy and delineation of potential target rocks. On the other hand, the weight and price of current SWIR sensors make it challenging to use them in practice. However, with increasing demand and technological innovation, it is expected that these HSI sensors will rapidly become cheaper and lighter and have an increased role in exploration. UAVs can also serve as platforms for a large amount of other sensors such as LIDAR^32,33^, magnetometers^34^ or radiometers^35^. The value of HSI can thus greatly be enhanced with the integration of additional remote sensing and geophysical data^13^. We expect drone-borne HSI to become standard in exploration schemes.

## Conclusion

We were able for the first time to directly identify and map REEs in carbonatite outcrops in both arid and subarctic environments using UAV-based hyperspectral data. The flexibility of UAV-based hyperspectral imaging can ensure personal safety as well as efficiency and speed during the exploration process. Areas previously inaccessible can now be rapidly surveyed, timely providing a more complete data set in terms of geological and topographical information. Current challenges in identifying REEs however can be overcome with diligent field planning, robust processing methods and new developments in sensor technology. In addition, using UAV-based hyperspectral imaging opens doors to new possibilities such as re-assessing REE occurrences in tailing dumps as possible resources for future beneficiation methods. This work validates the predictions made by Neave et al.^9^ and Zimmermann et al.^4^ concerning the sub-metre mapping of REEs capabilities of UAV-HSI. Ultimately, this study provides the opportunity to advance the discovery of REE deposits in most parts of the world.

## Methods

We surveyed two sites in southern Namibia and in central Finland to assess the REE potential of the deposits. The parameters of the two types of UAVs used as well as the attached sensor information can be found in Table 3. Nadir-stereo photos were captured with an RGB camera from the fixed-winged UAS with a 85% forward overlap and 70% side overlap. We produced the DEMs and orthomosaics using Agisoft PhotoScan Professional 1.2.5 in a standard SfM-MVS work flow. This is done by following protocols set out by previous studies^36,37^.

**Table 3:** UAS and data parameters of field surveys.

UAS	Mounted sensor	Parameters of imagery	Spatial resolution	Purpose
Multi-copter (Tholeg THO-R-PX-8/10)	Senop Hyperspectral Imager (Rikola)	50 spectral channels in aerial mode. Spectral sampling of 8 nm at Siilinjärvi and 5 nm at Marinkas Quellen. Spectral range from 500 - 900 nm. Integration time 10 ms	6.5 cm at 100 m altitude.	High resolution aerial HSI
Fixed-wing (SenseFly eBeePlus)	Sensefly S.O.D.A.	Nadir stereo-photos	2.9 cm at 120 m altitude	Photogrammetry for topographic information

We selected exposed outcrops for the hyperspectral surveys. We acquired the data with a UAV-mounted Rikola imager stabilized with a gimbal in a stop-and-go procedure (see Jakob et al.^20^ for details) at an altitude of 20 m at Marinkas Quellen and 40 m at at Siilinjärvi. These flight parameters resulted in a GSD of 1,5 cm at Marinkas Quellen and 3 cm at Siilinjärvi. We selected two different flight altitudes in order to test the REE mapping capabilities with varying pixel sizes. The integration time of the sensor at both sites was set to 10 ms and the signal-to-noise ratio (SNR) of the sensor is 150:1^10^. We selected spectral sampling intervals of 5 nm at Marinkas Quellen and 8 nm at Siilinjärvi with a spectral resolution of 15 nm.

**Figure 7:**
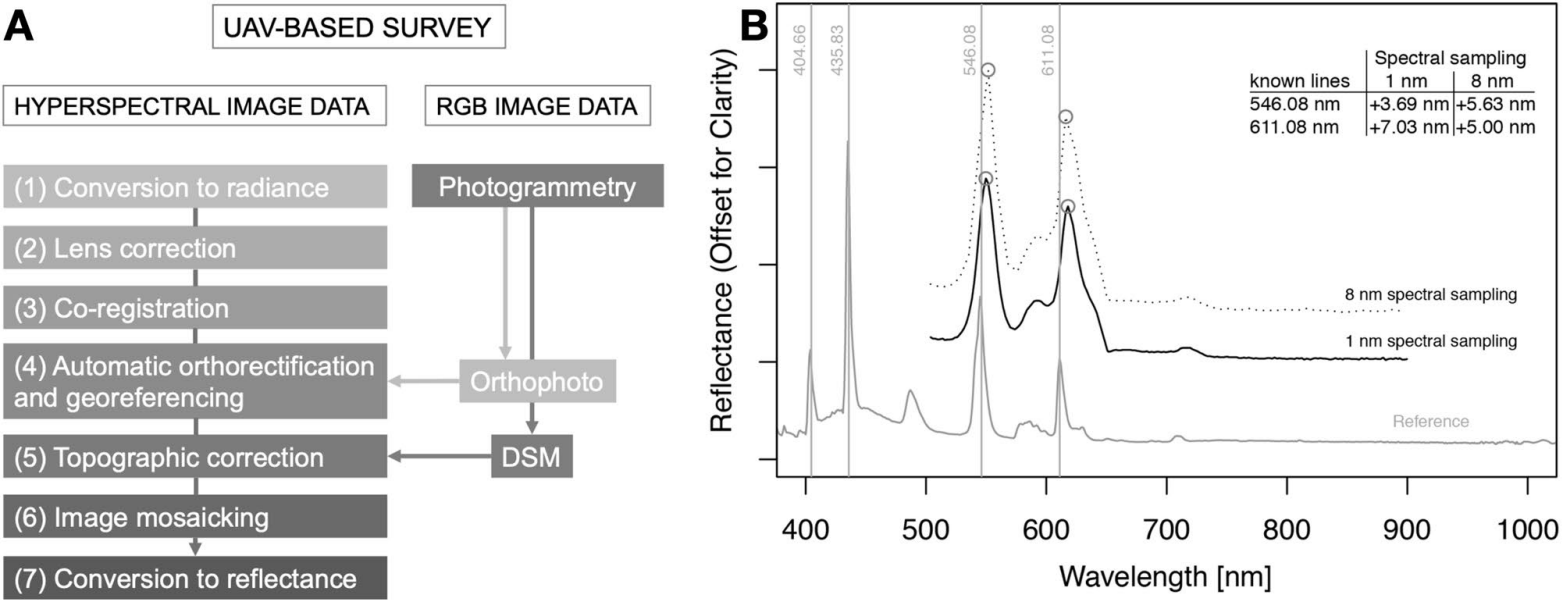
(**A**) Pre-processing steps of UAV-based hyperspectral data^20^. (**B**) Spectral check of the RIKOLA hyperspectral imager with 1 nm and 8 nm spectral sampling respectively compared to a reference fluorescent lamp with known emissions of Hg (404.66 nm, 435.83 nm and 546.08 nm) and Ba (611.08 nm).

The hyperspectral data is calibrated and corrected using an established python-based, in-house routine and can be seen in Fig. 7 A^20^. The routine contains 7 steps including spectral- and geometric corrections:

1. We performed a dark current subtraction on single images and the raw digital numbers were converted to radiance.
2. The internal camera features causes specific lens distortions and were corrected using the in-house toolbox.
3. During image acquisition the sensor would slightly move, causing spatial shifts between the individual bands. To rectify this, we co-registered the single bands with one another for each image.
4. We automatically orthorectified and georeferenced the hyperspectral images. In order to do this we used keypoint detection and point-matching algorithms to match the hyperspectral data with the SfM-MVS generated orthomosaics
5. As terrain roughness also influences the measured radiance, we used the high-resolution DSMs, which were produced from SfM-MVS photogrammetry, to calculate the incidence angle of the incoming light for topographic corrections.
6. We then stitched together the individually corrected hyperspectral images to produce a mosaic.
7. We applied an empirical line correction by using known spectra from black, white, and grey polyvinyl chloride (PVC) panels placed in the scene. Lastly, we masked out the vegetation in the entire mosaic.

The final product is a calibrated, illuminatively and geometrically corrected hyperspectral mosaic in reflectance. We captured the data during peak sunlight conditions and focused on exposed outcrops without any shadows. Additionally, the influence of the atmosphere is almost insignificant due to the low acquisition altitudes, thus atmospheric corrections were not needed.

Prior to field work, the potential of the Rikola imager to resolve REEs was evaluated in laboratory conditions^3^. This showed that a spectral shift in the Rikola spectra of about 5 nm in flight mode (50 bands, 8nm spectral sampling) is present and should be corrected for. Further, a routine spectral check using a fluorescent lamp with known emission lines of Hg and Ba was performed. It should also be noted that the shift differs for the two sensors of the Rikola (in particular visible in the 1 nm spectral sampling) (Fig. 7B).

**Figure 8:**
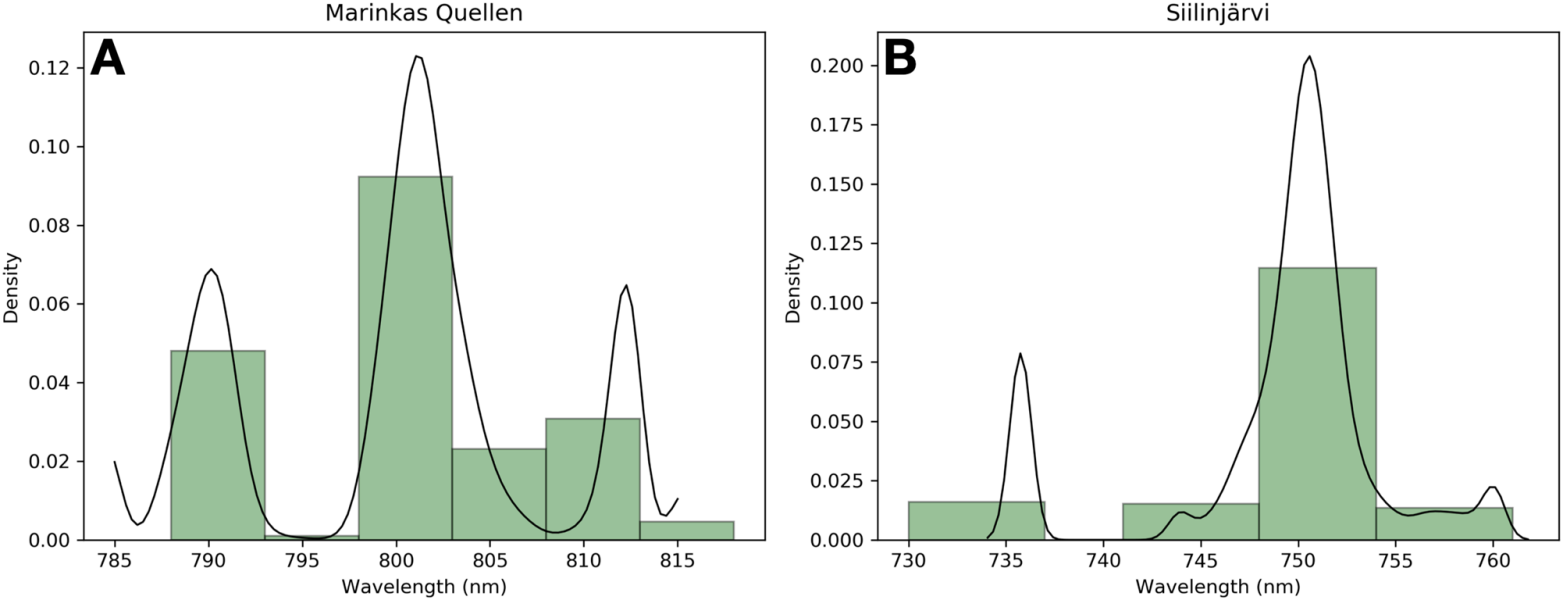
(**A**) Density distribution function and histogram of pixels mapped at 800 nm from Marinkas Quellen. Size of bins are based on the spectral sampling of 5 nm. (**B**) Density distribution function and histogram of pixels mapped at 750 nm from Siilinjärvi. Size of bins are based on the spectral sampling of 8 nm.

After pre-processing the data, we map the characteristic absorption features using the minimum wavelength mapping (MWM) method with a polynomial function^38^ taking into account the spectral shift observed in the laboratory results. This method directly determines the wavelength position of the deepest absorption features in a specified spectral range and its depth^38^. The result is a map highlighting pixels that have a specific absorption position while the color (in the present case from yellow to red) indicates the increasing depth of the absorption feature. To validate the results we plot a histogram of mapped pixels at the aforementioned wavelengths from each data set. In the case for Marinkas Quellen and Siilinjärvi, we plot histograms of pixels mapped at 800 nm and 750 nm, respectively (Fig. 8A,B). In both cases most pixels were mapped at the respective wavelengths, indicating that the mapped absorption features at 750 nm and 800 nm are not random and are indeed significant.

During the field campaign we collected rock samples to validate the hyperspectral data. In-situ spectral measurements were taken with the Spectral Evolution PSR-3500 VNIR-SWIR spectrometer while laboratory whole rock geochemical analyses were performed on the rock samples. A Global Positioning System (GPS) and detailed field photos were used to match the position of the measurements and rock samples with their locations on the UAV-based imagery. ICP-MS was used to analyse the trace elements of the samples at both study areas. Wavelength dispersive X-ray fluorescence (WD-XRF) was used to analyse the major elements for the samples at Marinkas Quellen while Inductively coupled plasma atomic emission spectroscopy (ICP-AES) was used to analyse the major elements of rocks from Siilinjärvi. Tables 4 and 5 show the geochemical results of the samples taken at Marinkas Quellen and Siilinjärvi.

**Table 4:** Whole rock geochemical analyses of major elements in wt% for Marinkas Quellen (Mar) and Siilinjärvi (Siil).

Samples	(Mar) Point 1	(Mar) Point 2	(Mar) Point 3	(Mar) Point 4	(Mar) Point 5	(Mar) Point 6	(Mar) Point 7	(Siil) Point A	(Siil) Point B	(Siil) Point C
$SiO_{2}$	1.92	4.76	4.15	0.13	0.29	4.01	0.98	38.4	1.75	0.58
$Al_{2}O_{3}$	0.24	0.83	0.81	0.06	0.09	1.10	0.27	8.55	0.41	0.13
$Fe_{2}O_{3}$	1.25	2.78	3.89	1.75	0.88	4.83	9.35	8.53	1.55	0.88
*MgO*	0.45	2.72	4.76	3.89	0.91	7.84	6.39	22.2	6.18	3.34
*CaO*	51.64	46.39	42.17	47.00	52.17	38.15	39.10	5.08	48.1	52.3
$P_{2}O_{5}$	0.14	2.18	2.79	1.03	0.12	1.62	0.04	3.2	2.02	14.35

**Table 5:** Whole rock geochemical analyses of rare earth minor elements in ppm for Marinkas Quellen (Mar) and Siilinjärvi (Siil).

Samples	(Mar) Point 1	(Mar) Point 2	(Mar) Point 3	(Mar) Point 4	(Mar) Point 5	(Mar) Point 6	(Mar) Point 7	(Siil) Point A	(Siil) Point B	(Siil) Point C
La	274	278	467	352	173	1340	12.7	43.1	127	227
Ce	521	619	991	700	321	2030	30.6	120.5	316	613
Pr	53.8	68.3	110.5	70.7	34.9	193.5	4.19	16.45	39.5	80.5
Nd	225	278	443	261	129.5	647	18.6	67	156.5	325
Sm	53,7	45.5	69.1	38.4	19.95	63.3	4.7	9.52	23.7	48
Eu	18.65	13.7	19.75	11.1	6.12	15.8	1.63	2.51	6.01	12.65
Gd	50.9	36.9	49.7	28.8	1.4	35.2	5.78	5.6	14.95	29.5
Tb	6.29	4.15	5.74	3.66	1.97	4.09	1.46	0.57	1.66	3.03
Dy	31.8	20.6	27.9	18.5	9.79	18.7	11.9	2.27	7.51	12.65
Ho	5.36	3.47	4.66	3.45	1.86	3.1	2.82	0.31	1.15	1.87
Er	12.35	7.97	10.55	8.05	4.6	6.99	9.15	0.55	2.65	3.54
Tm	1.64	0.98	1.18	1.12	0.66	0.95	1.47	0.05	0.3	0.4
Yb	10.15	5.84	7.19	6.92	3.82	5.4	11.25	0.25	1.54	1.91
Lu	1.4	0.78	0.98	1.09	0.55	0.75	1.74	0.04	0.22	0.24
Y	135	87.2	114	90.1	48.5	79.2	88.2	7.6	32.3	44.1
TREO (%)	0.16	0.18	0.29	0.20	0.09	0.57	0.02	0.03	0.09	0.18

## References

[1] Simandl G (2014). Geology and market-dependent significance of rare earth element resources. Miner. Depos..

[2] Schmid M (2019). Rare earths in the trade dispute between the US and China: A déjà vu. Intereconomics.

[3] Booysen R (2019). Towards multiscale and multisource remote sensing mineral exploration using rpas: A case study in the lofdal carbonatite-hosted ree deposit, Namibia. Remote Sens..

[4] Zimmermann R, Brandmeier M, Andreani L, Mhopjeni K, Gloaguen R (2016). Remote sensing exploration of nb-ta-lree-enriched carbonatite (Epembe/Namibia). Remote Sens..

[5] Goodenough, K. *et al.* Europe’s earth element resource potential: An overview of ree metallogenetic provinces and their geodynamic setting. *Ore Geol. Rev.* **72**, 838–856. 10.1016/j.oregeorev.2015.09.019 (2016).

[6] Wall F (2014). Rare Earth Elements.

[7] Simandl GJ, Paradis S (2018). Carbonatites: Related ore deposits, resources, footprint, and exploration methods. Appl. Earth Sci..

[8] Van Gosen, B. S., Verplanck, P. L., Long, K. R., Gambogi, J. & Seal, R. R. II. *The Rare-Earth Elements: Vital to Modern Technologies and Lifestyles* (Technical Report, US Geological Survey, 2014).

[9] Neave DA (2016). On the feasibility of imaging carbonatite-hosted rare earth element deposits using remote sensing. Econ. Geol..

[10] Lorenz S (2019). The potential of reflectance and laser induced luminescence spectroscopy for near-field rare earth element detection in mineral exploration. Remote Sens..

[11] Turner DJ, Rivard B, Groat LA (2016). Visible and short-wave infrared reflectance spectroscopy of ree phosphate minerals. Am. Mineral..

[12] Boesche NK (2016). Hyperspectral rare earth element mapping of three outcrops at the fen complex, norway: Calcitic, dolomitic, and ankeritic carbonatites. Rare Earths Ind..

[13] Jackisch R (2019). Drone-borne hyperspectral and magnetic data integration: Otanmäki fe-ti-v deposit in finland. Remote Sens..

[14] Malehmir A (2017). The potential of rotary-wing UAV-based magnetic surveys for mineral exploration: A case study from central sweden. Lead. Edge.

[15] Sayab M, Aerden D, Paananen M, Saarela P (2018). Virtual structural analysis of jokisivu open pit using ‘structure-from-motion’unmanned aerial vehicles (UAV) photogrammetry: Implications for structurally-controlled gold deposits in southwest Finland. Remote Sens..

[16] Katuruza M, Birch C (2019). The use of unmanned aircraft system technology for highwall mapping at Isibonelo Colliery, South Africa. J. S. Afr. Inst. Min. Metall..

[17] Kirsch M (2018). Integration of terrestrial and drone-borne hyperspectral and photogrammetric sensing methods for exploration mapping and mining monitoring. Remote Sens..

[18] Madjid M, Vandeginste V, Hampson G, Jordan C, Booth A (2018). Drones in carbonate geology: Opportunities and challenges, and application in diagenetic dolomite geobody mapping. Mar. Petrol. Geol..

[19] Dujoncquoy, E. *et al.* Uav-based 3d outcrop analog models for oil and gas exploration and production. In *IGARSS 2019–2019 IEEE International Geoscience and Remote Sensing Symposium*, 6791–6794 (2019).

[20] Jakob S, Zimmermann R, Gloaguen R (2017). The need for accurate geometric and radiometric corrections of drone-borne hyperspectral data for mineral exploration: Mephysto–a toolbox for pre-processing drone-borne hyperspectral data. Remote Sens..

[21] Bernasconi, A. The Marinkas kwela alkali intrusive: A porphyry molybdenum system of Cambrian age in Southern South West Africa/Namibia. In *Mineral Deposits of Southern Africa* 1587–1591 (1986).

[22] Smithies R, Marsh J (1998). The Marinkas Quellen Carbonatite Complex, southern Namibia: Carbonatite magmatism with an uncontaminated depleted mantle signature in a continental setting. Chem. Geol..

[23] Rukhlov A, Bell K (2010). Geochronology of carbonatites from the Canadian and Baltic Shields, and the Canadian Cordillera: Clues to mantle evolution. Mineral. Petrol..

[24] O’Brien, H., Heilimo, E. & Heino, P. The archean siilinjärvi carbonatite complex. In *Mineral Deposits of Finland* 327–343 (2015). 10.1016/B978-0-12-410438-9.00013-3.

[25] Al-Ani, T. Mineralogy and petrography of Siilinjärvi carbonatite and Glimmerite rocks, eastern Finland. In *Geological Survey of Finland Report*, vol. 164 (2013).

[26] Geological Survey of Finland. Bedrock map of Finland scale-free (2017). https://hakku.gtk.fi/en. Last accessed 10 Dec 2019.

[27] Tommaselli A, Santos L, Berveglieri A, Oliveira R, Honkavaara EA (2018). A study on the variations of inner orientation parameters of a hyperspectral frame camera. Int. Arch. Photogramm. Remote. Sens. Spatial Inf. Sci..

[28] Rowan LC, Kingston MJ, Crowley JK (1986). Spectral reflectance of carbonatites and related alkalic igneous rocks; selected samples from four North American localities. Econ. Geol..

[29] Aasen H, Honkavaara E, Lucieer A, Zarco-Tejada PJ (2018). Quantitative remote sensing at ultra-high resolution with uav spectroscopy: A review of sensor technology, measurement procedures, and data correction workflows. Remote Sens..

[30] Haldar, S. *Mineral Exploration*, 2nd edn, 69–84 (Elsevier, 2018).

[31] Demetriades A, Smith D, Wang X (2019). General concepts of geochemical mapping at global, regional, and local scales for mineral exploration and environmental purposes. Geochim. Bras..

[32] Wallace, L., Lucieer, A. & Watson, C. Assessing the feasibility of uav-based lidar for high resolution forest change detection. In *The 12th Congress of the International Society for Photogrammetry and Remote Sensing*, vol. 39, 499–504 (2012).

[33] Chisholm RA, Cui J, Lum SK, Chen BM (2013). UAV LiDAR for below-canopy forest surveys. J. Unmanned Veh. Syst..

[34] Gavazzi B, Le Maire P, Munschy M, Dechamp A (2016). Fluxgate vector magnetometers: A multisensor device for ground, uav, and airborne magnetic surveys. Lead. Edge.

[35] Šálek O, Matolín M, Gryc L (2018). Mapping of radiation anomalies using UAV mini-airborne gamma-ray spectrometry. J. Environ. Radioact..

[36] Carrivick JL, Smith MW, Quincey DJ (2016). Structure from Motion in the Geosciences.

[37] James MR, Robson S, d’Oleire Oltmanns S, Niethammer U (2017). Optimising UAV topographic surveys processed with structure-from-motion: Ground control quality, quantity and bundle adjustment. Geomorphology.

[38] Van Ruitenbeek FJ (2014). Mapping the wavelength position of deepest absorption features to explore mineral diversity in hyperspectral images. Planet. Space Sci..

